# Vulvar dermatoses: a cross-sectional 5-year study. Experience in a specialized vulvar unit^☆^

**DOI:** 10.1016/j.abd.2021.11.006

**Published:** 2022-09-08

**Authors:** Fernando García-Souto, Ana Isabel Lorente-Lavirgen, Francisco Manuel Ildefonso Mendonça, Manuel García-de-Lomas, Mariana Viktoria Hoffner-Zuchelli, Desiree Rodriguez-Ojeda, Elena Pozo, José Bernabéu-Wittel

**Affiliations:** a Dermatology Department, Hospital Viamed Santa Angela de la Cruz, Seville, Spain; b Gynecology and Obstetrics Department, Hospital Viamed Santa Angela de la Cruz, Seville, Spain; c Fisinergia Fisioterapia Pelvico-Perineal, Seville, Spain

**Keywords:** Genital disease, Pelvic pain, Vulvar diseases, Vulvodynia

## Abstract

**Background:**

Vulvar diseases are common in the general population and have a negative impact on the quality of life.

**Objectives:**

To describe our experience as dermatologists in the management of vulvar dermatosis consultations.

**Methods:**

A retrospective observational study was conducted with patients who attended monographic vulvar consultations over a 5-year period. Clinical information was obtained from the patient’s charts.

**Results:**

148 women were studied. Their mean age was 43.24 years (standard deviation: 15.15 years), with ages ranging from 4 months to 80 years. 53.4% of patients took between 2 and 5 years to seek medical attention for the first time. The most frequent diagnosis was lichen sclerosus (41.9%), irritative eczema of the vulva (14.9%), and lichen simplex chronicus (10.1%). 83.8% reported anogenital itching, 66.2% pain, and 45.9% dyspareunia. The most frequently prescribed treatment was ultra-potent topical corticosteroids (clobetasol propionate; 41.2%). Patients with lichen sclerosus were significantly older than those who presented with any of the other diseases. No differences were found in terms of either the time of disease evolution or in symptom presentation.

**Study limitations:**

Retrospective study. Vulvar diseases with an infectious cause are usually managed in primary care, therefore, were not included. All patients were recruited from a single private hospital which limits the comparisons with the public health system.

**Conclusions:**

Vulvar diseases frequently occur and are associated with high morbidity. It is essential to promote the development of specific vulvar consultations in hospitals. Specialties such as dermatology, gynecology, urology, or physiotherapy must be part of these units.

## Introduction

Vulvar diseases are common in the general population, although their true importance and frequency are often underestimated.^1^ Most vulvar diseases have a markedly negative impact on patients’ quality of life.^2^, ^3^ Whether due to ignorance or shame, the vulva is an area of the body that is usually neglected, both by patients and professionals. In addition, vulvar diseases are typically associated with significant diagnostic delays, as patients with vulvar symptoms usually take a long time to see a doctor.^4^

Even though gynecologists are specialists who are generally the first to see patients with vulvovaginal symptoms, they generally receive little training in the management of dermatological diseases of the vulva.^4^ Dermatologists are experts in the evaluation and management of the skin and mucous membranes, including the genitals. However, the vulva is an area that is often overlooked by dermatologists. The most common reasons for this include a lack of training, discomfort during the clinical interview, and ignorance of the complementary tests commonly used in vulvar disorders.^4^ In a survey carried out in the United States that aimed to evaluate competences in the management of vulvar diseases among medical students, and dermatology and gynecology residents, only 19% of those surveyed reported having received specific training during residency.^5^

Although there has been a growing interest among dermatologists in the study of the vulva in recent years^6^, ^7^ – particularly among dermatologists in the United Kingdom, which is one of the leading countries to develop specific units dedicated to treating diseases of the vulva – there are currently few scientific studies on the matter. Consequently, the objective of this study is to describe our experience as dermatologists in the management of vulvar dermatosis consultations.

## Materials and methods

A retrospective observational study was carried out over a 5-year period in Viamed Santa Angela de la Cruz hospital in Seville, southern Spain. Between May 2015 and May 2020, all patients who visited our monographic vulvar unit were included in the study. Data collection was carried out by a specialist in dermatology. The data were obtained from the clinical history of each patient. The following variables were collected for analysis: patients’ age, symptoms, time of evolution, main and secondary diagnoses, complementary tests, and treatments used. The study was approved by the ethics committee of the hospital, and a study information document was provided to patients who were interested in participating in the study.

A descriptive analysis was performed using measures of central tendency and dispersion for quantitative variables, and frequency distribution for qualitative variables. For the statistical analysis, the authors used the statistical Chi-Squared test, or Fisher’s exact test if the expected frequencies were <5, in order to compare the qualitative variables. The Mann–Whitney *U* test was used to compare the quantitative variables. The level of statistical significance was established at α = 0.05. Statistical analyses were performed with SPSS version 25.0 for Apple macOS (IBM Corporation, Armonk, NY, USA).

## Results

A total of 148 women were studied at our monographic vulva clinic over a 5-year period. The mean ± Standard Deviation age (SD) was 43.24 ± 15.15 years, with patients’ ages ranging from 4 months of age to 80 years. Patients were divided by age with the following distribution: <18 years (n = 10; 6.8%), between 18 and 40 years (n = 50; 33.8%), between 40 and 60 years (n = 68; 45.9%), and >60 years (n = 20; 13.5%).

The most frequent primary diagnosis was lichen sclerosus (n = 62; 41.9%), followed by irritative eczema of the vulva (n = 22; 14.9%), and lichen simplex chronicus (n = 15; 10.1%). A total of 14 (9.5%) patients also had another concomitant dermatosis at the time of the visit. The evolution of the different primary diseases was as follows: <1 year (n = 41; 27.7%), between 2 and 5 years (n = 79; 53.4%), and >5 years (n = 28; 18.9%). A total of 124 patients (83.8%) reported anogenital itching, 98 (66.2%) pain, and 68 (45.9%) dyspareunia. Some complementary tests were performed in 36 (22.3%) cases, of which 14 (9.5%) had microbiological samples taken, and 22 (14.9%) had a skin biopsy performed.

The most frequently prescribed treatment was ultra-potent topical corticosteroids (clobetasol propionate; n = 61; 41.2%) and the most commonly prescribed adjuvant treatment was topical ketoconazole gel (n = 42; 28.4%). In 34 (23%) women, pelvic floor physiotherapy was recommended for vulvar discomfort, in 23 (15.5%) antihistamines, and in 15 (10.1%) a formula with 0.5% triamcinolone. In 8 (5.4%), calcineurin inhibitors were prescribed. Other prescribed treatments were cryotherapy (n = 4; 2.7%), topical antifungals (n = 15; 10.1%), topical and/or oral antibiotics (n = 7; 4.7%), vaginal dilators (n = 1; 0.7%), topical imiquimod (n = 2; 1.4 %), topical gabapentin (n = 1; 0.7%), topical diltiazem (n = 1; 0.7%), antivirals (n = 4; 2.7%), and surgery (n = 2; 1.4%).

**Figure 1:**
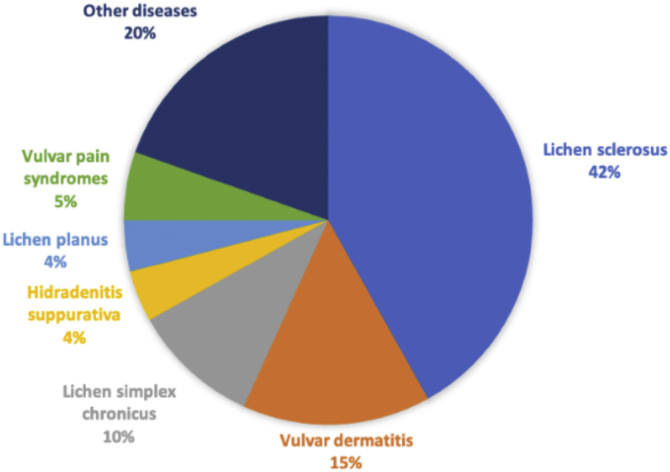
Percentage of the different diagnoses identified during the study.

**Figure 2:**
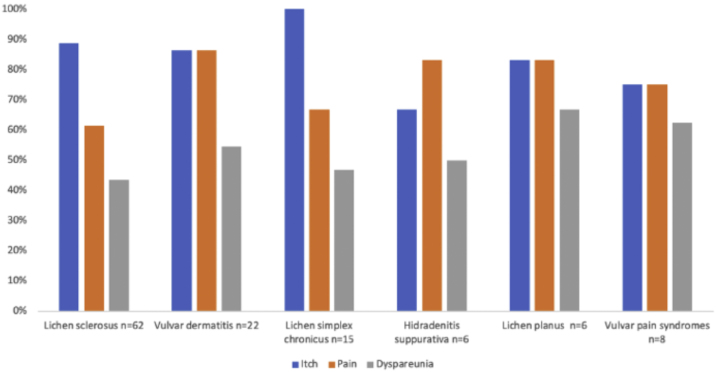
Percentages of itch, pain, and dyspareunia for each of the most relevant diagnoses identified during the study.

A detailed analysis of the different diagnoses collected is represented in Table 1 and Fig. 1. The different features of the most relevant diseases identified during this study are shown in Table 2, Table 3. Fig. 2 shows the proportion of pain, itching, and dyspareunia, which represent the main diseases identified in this work.

**Table 1:** List of primary and secondary diagnoses collected during the study period.

Primary diagnosis	n (%)
Lichen sclerosus	62 (41.9)
Irritant eczema	22 (14.9)
Lichen simplex chronicus	15 (10.1)
Hidradenitis suppurativa	6 (4.1)
Lichen planus	6 (4.1)
Provoked secondary vestibulodynia	6 (4.1)
Recurrent vulvo-vaginal candidiasis	4 (2.7)
Candidal intertrigo	3 (2)
Vulvar fissure	3 (2)
Condylomas	3 (2)
Vulvar melanosis	2 (1.4)
Vulvodynia	2 (1.4)
Vestibular spicules	2 (1.4)
Paget’s disease of the vulva	1 (0.7)
Fordyce glands	1 (0.7)
Leukoplakia	1 (0.7)
Episiotomy dehiscence	1 (0.7)
Genito-urinary syndrome	1 (0.7)
Normal vulva	1 (0.7)
Sebocystomas	1 (0.7)
Anal fissure	1 (0.7)
Clitoral hyperesthesia	1 (0.7)
Common VIN	1 (0.7)
Scleroderma	1 (0.7)
Genital herpes	1 (0.7)
TOTAL	148 (100)

Secondary diagnoses	n (%)
None	134 (90.5)
Secondary candidiasis	2 (1.4)
Candidal intertrigo	2 (1.4)
Condylomas	1 (0.7)
Irritative eczema	1 (0.7)
Perianal eczema	1 (0.7)
Vulvar fissure	1 (0.7)
Lichen sclerosus	1 (0.7)
Lichen planus	1 (0.7)
Molluscum	1 (0.7)
Differentiated VIN	1 (0.7)
Vitiligo	1 (0.7)
TOTAL	148 (100)

VIN, Vulvar Intraepithelial neoplasia.

**Table 2:** Demographic characteristics, evolution, symptoms, complementary tests, and treatments of patients with lichen sclerosus when compared with the rest of the vulvar dermatoses collected in the study.

	Lichen sclerosus (n = 62)	Other dermatoses (n = 86)	p
**Age (years)**	46.33 ± 2.33	41.01 ± 1.29	**0.002**
	**n (%)**	**n (%)**	
**Evolution (years)**			
<1	13 (21)	28 (32.6)	0.186
2–5	34 (54.8)	45 (52.3)	
>5	15 (24.2)	13 (15.1)	
**Symptoms**			
Itching	55 (88.7)	69 (80.2)	0.167
Pain	38 (61.3)	60 (69.8)	0.282
Dyspareunia	27 (43.5)	41 (47.7)	0.619
**Supplementary tests**			
Microbiology	4 (6.5)	10 (11.6)	0.288
Skin biopsy	13 (15.1)	9 (14.5)	0.919
**Treatments**			
Ultra-potent topical corticosteroid	45 (72.6)	16 (18.6)	**<0.001**
Topical calcineurin inhibitors	6 (9.7)	0 (0)	**0.005**
Pelvic floor physiotherapy	9 (14.5)	25 (29.1)	**0.038**
Triamcinolone 0.5% gel	8 (12.9)	7 (8.1)	0.412
**Adjuvant treatments**			
Topical ketoconazole	25 (40.3)	17 (19.8)	**0.006**
Antihistamines	9 (14.5)	14 (16.3)	0.770

Bold values signifies the values that have statistical significance (p < 0.05).

**Table 3:** Demographic characteristics, evolution, symptoms, complementary tests, and treatment of some of the most frequently identified diagnoses in the study.

	Irritant eczema of vulva (n = 22)	Lichen simplex chronicus (n = 15)	Hidradenitis suppurativa (n = 6)	Lichen planus (n = 6)	Vulvar pain syndromes (n = 8)
**Age (years)**	38.19 ± 15.41	41.51 ± 7.00	45.03 ± 7.69	56.41 ± 13.22	40.71 ± 11.09
	**n (%)**	**n (%)**	**n (%)**	**n (%)**	**n (%)**
**Evolution (years)**					
<1	5 (22.7)	7 (46.7)	1 (16.7)	1 (16.7)	1 (12.5)
2–5	15 (68.2)	5 (33.3)	3 (50)	5 (83.3)	3 (37.5)
>5	2 (9.1)	3 (20)	2 (33.3)	0 (0)	4 (50)
**Symptoms**					
Itch	19 (86.4)	15 (100)	4 (66.7)	5 (83.3)	6 (75)
Pain	19 (86.4)	10 (66.7)	5 (83.3)	5 (83.3)	6 (75)
Dyspareunia	12 (54.5)	7 (46.7)	3 (50)	4 (66.7)	5 (62.5)
**Supplementary tests**					
Microbiology	3 (13.6)	1 (6.7)	0 (0)	1 (16.7)	1 (12.5)
Skin biopsy	2 (9.1)	0 (0)	0 (0)	5 (83.3)	0 (0)
**Treatments**					
Ultra-potent topical corticosteroid	4 (18.2)	7 (46.7)	0 (0)	4 (66.7)	0 (0)
Topical calcineurin inhibitors	0 (0)	0 (0)	0 (0)	0 (0)	0 (0)
Pelvic floor physiotherapy	5 (22.7)	2 (13.3)	1 (16.7)	3 (50)	6 (75)
Triamcinolone 0.5% gel	1 (4.5)	2 (13.3)	0 (0)	4 (66.7)	0 (0)
Oral and/or topical antibiotics	0 (0)	0 (0)	3 (50)	0 (0)	0 (0)
Surgery	0 (0)	0 (0)	0 (0)	0 (0)	1 (12.5)
Psychotherapy	0 (0)	0 (0)	0 (0)	0 (0)	1 (12.5)
**Adjuvant treatments**					
Topical ketoconazole	5 (22.7)	6 (40)	1 (16.7)	1 (16.7)	0 (0)
Antihistamines	5 (22.7)	6 (40)	0 (0)	0 (0)	0 (0)

The authors described the patients’ characteristics based on the most relevant diagnoses. The authors performed a comparative analysis between those patients who presented with lichen sclerosus sclerosis versus those who presented with other vulvar dermatoses.

### Lichen sclerosus (Fig. 3)

In all, 62 patients with lichen sclerosus were studied; patients had a mean ± SD age of 46.33 ± 18.35 years. The evolution was <1 year in 13 (21%) patients, between 2–5 years in 34 (54.8%), and >5 years in 15 (24.2%). A second concomitant dermatosis was recorded in 7 patients, with vitiligo being the most frequent (n = 2; 3.2%). Overall, 55 (88.7%) patients had itching, 38 (61.3%) pain, and 27 (43.5%) dyspareunia. A skin biopsy was taken in 9 (14.5%) of the cases and microbiological samples were obtained in 4 (6.5%). Skin biopsies were taken to confirm lichen sclerosus diagnosis in clinically doubtful cases, in steroids-resistant cases (Fig. 4) and in ulcerated or hyperkeratotic lesions in order to rule out malignancy (Figure 5, Figure 6).

**Figure 3:**
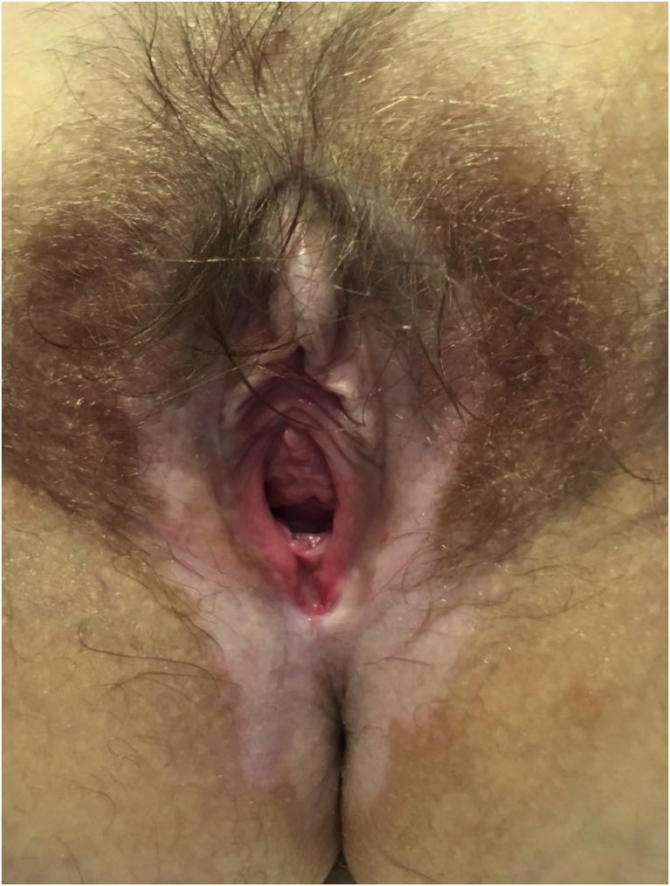
Lichen sclerosus.

**Figure 4:**
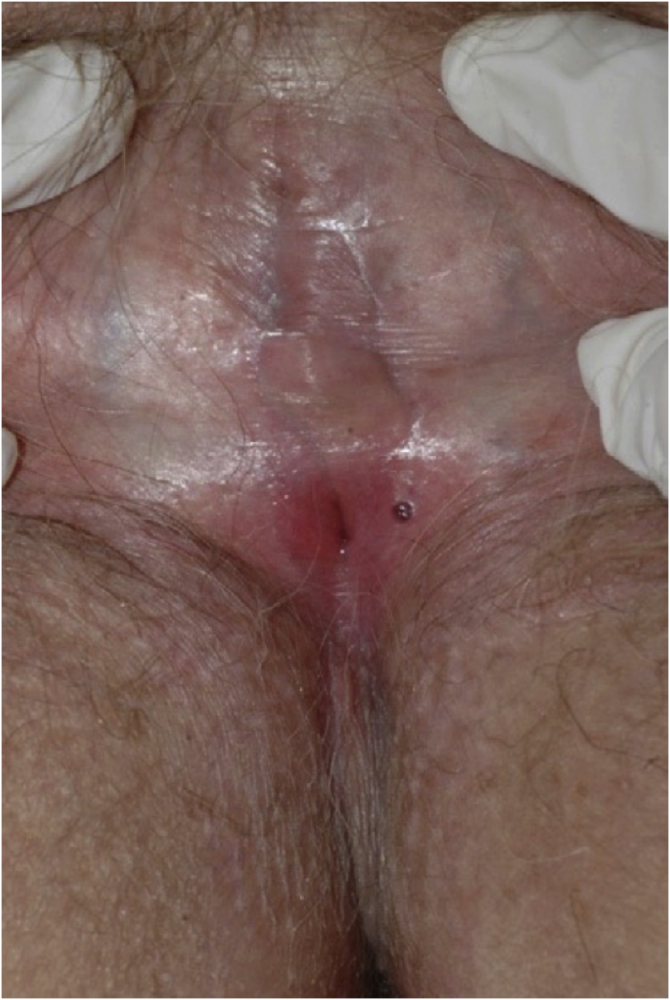
Advanced lichen sclerosus. Complete fusion of labia mayora.

**Figure 5:**
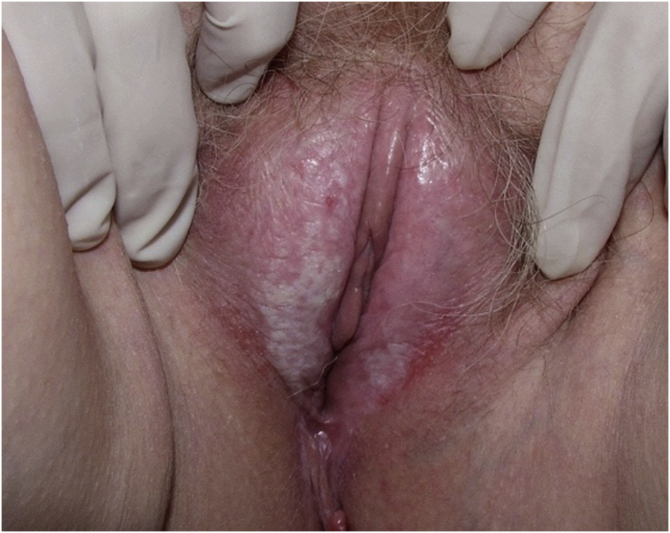
Paget's disease of the vulva.

**Figure 6:**
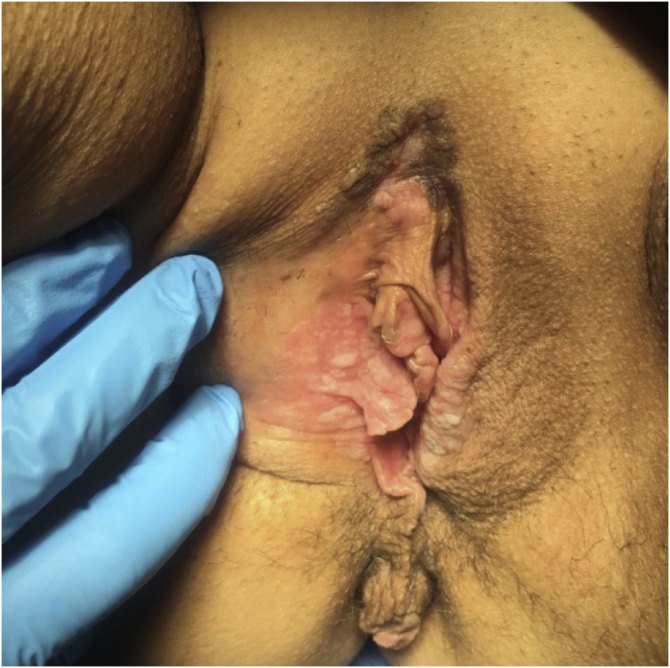
Common vulvar intraepithelial neoplasia (VIN).

The most frequent treatment was ultra-potent topical corticosteroids (clobetasol propionate; n = 45; 72.6%), with 2% ketoconazole gel serving as adjunctive therapy in 25 (40.3%) cases. In 8 (12.95%) patients, a formula with 0.5% triamcinolone in the gel base was used. In 9 (14.5%) women, antihistamines were also indicated for itching.

A comparative analysis was performed between the patients with lichen sclerosus, and the rest of the patients included in the study. Statistically significant differences were found in terms of age and some of the prescribed treatments (ultra-potent topical corticosteroids, calcineurin inhibitors, pelvic floor physiotherapy, and topical ketoconazole). No differences were found either in terms of the time of evolution of the disease or in the symptoms. Table 2 shows the characteristics of patients with lichen sclerosus and provides a comparative analysis featuring the remaining cases.

### Irritant eczema of the vulva (Fig. 7)

Twenty-two patients with irritative vulvar eczema were studied; they had a mean ± SD age of 38.19 ± 15.41 years being the younger one a 4-month-old girl. The evolution was <1 year in 5 (22.7%) patients, from 2–5 years in 15 (68.2%), and >5 years in 2 (9.1%). In a single patient (the aforementioned 4-month-old patient a concomitant dermatosis was recorded: a candidal intertrigo. In all, 19 (86.4%) patients had itching, 19 (86.4%) pain, and 12 (54.5%) dyspareunia. A skin biopsy was taken in only 2 (9.1%) cases, and microbiological samples in 3 (13.6%). An ultra-potent topical corticosteroid was indicated in 4 (18.2) patients, pelvic floor physiotherapy in 5 (22.7%), and a formulation with 0.5% triamcinolone in 1 (4.5%). As adjunctive therapy, antihistamines were indicated in 5 (22.7%) cases, and ketoconazole 2% topical gel in 5 (22.7%).

**Figure 7:**
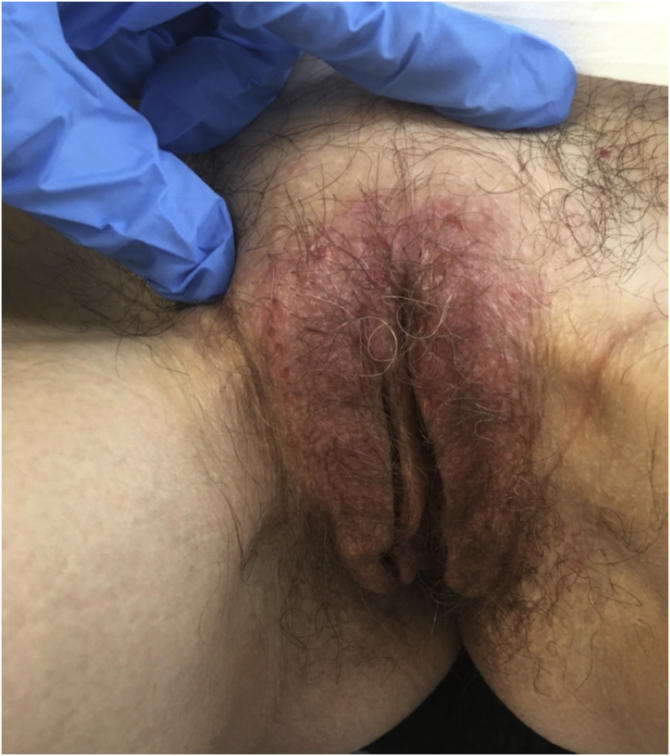
Irritant eczema of the vulva.

Among the products related to dermatitis, a wide variety of creams for intimate and general use were collected, the most frequent of which were blastostimulin and the use of intimate wipes for hygiene. In one case, treatment was related to the use of hair dye in a patient previously diagnosed with an allergy to p-phenylenediamine; this represents the only case of allergic contact dermatitis in our entire series.

### Chronic lichen simplex

Fifteen patients with lichen simplex chronicus were studied; they had a mean ± SD age of 41.51 ± 7.00 years. The most frequent evolution was <1 year in 7 (46.7%) patients. A concomitant dermatosis, candidal intertrigo, was recorded in a single patient. All patients had itching (100.0%), pain (n = 10; 66.7%), and dyspareunia (n = 7; 46.7%). In no cases was a skin biopsy taken, and microbiological samples were obtained in a single case. An ultra-potent topical corticosteroid was indicated in 7 (46.7%) patients, pelvic floor physiotherapy in 2 (13.3%), and a formula featuring 0.5% triamcinolone in 2 (13.3%). As adjunctive therapy, antihistamines were indicated in 6 (40%) cases, and topical ketoconazole in 6 (40%).

### Hidradenitis suppurativa (Fig. 8)

Six women with hidradenitis suppurativa were observed, with a mean ± SD age of 45.03 ± 7.69 years. The evolution was <1 year in 1 (16.7%) patient, between 2–5 years in 3 (50%), and >5 years in 2 (33.3%). Four (66.7%) patients had itching, 5 (83.3%) pain, and 7 dyspareunia (50%). In no cases were skin biopsies or microbiological samples taken. Topical and/or oral antibiotics were prescribed in three patients. Pelvic floor physiotherapy was indicated in one patient. As adjunctive therapy, 2% topical ketoconazole gel was indicated in one case.

**Figure 8:**
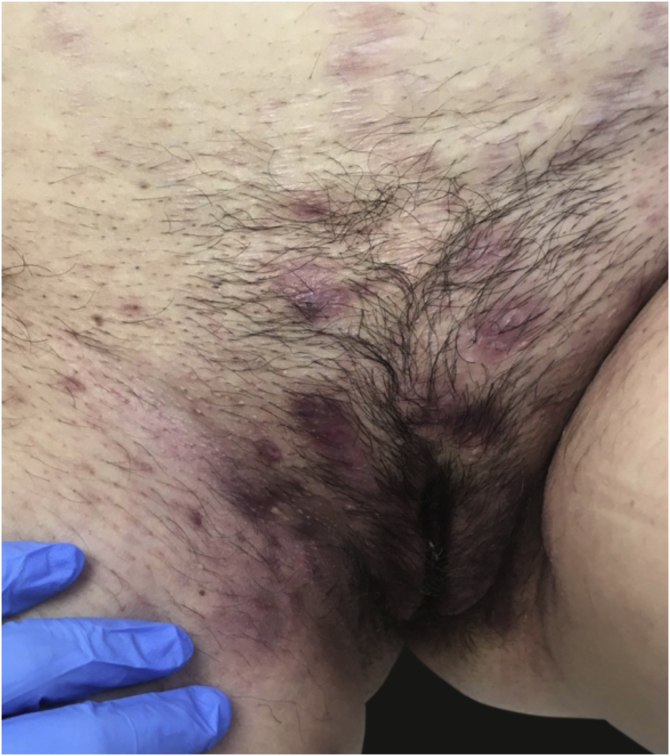
Hidradenitis suppurativa.

### Lichen planus

Six patients with vulvar lichen planus were evaluated, with a mean ± SD age of 56.41 ± 13.22 years. The most frequent evolution time was 2–5 years in 5 (83.3%) patients. Five (83.3%) patients had itching, 5 (83.3%) had pain, and 4 (66.7%) had dyspareunia. A skin biopsy was taken in 5 (83.3%) cases, and microbiological samples were obtained from a single patient. An ultra-potent topical corticosteroid was indicated in 4 (66.7%) patients; 0.5% triamcinolone was prescribed for 4 (66.7%) patients, and pelvic floor physiotherapy was indicated in 3 patients (50%). As adjunctive therapy, topical 2% ketoconazole gel was indicated in a single patient.

### Vulvar pain syndromes

#### Provoked secondary vestibulodynia

Eight patients attended our consultations for nonspecific vulvar pain, 6 of which were diagnosed with provoked secondary vestibulodynia, with a mean ± SD age of 44.06 ± 10.48 years. The evolution was <1 year in 1 (16.7%) patient, between 2–5 years in 3 (50%), and >5 years in 2 (33.3%). A concomitant dermatosis, vulvovaginal candidiasis, was recorded in a single patient. Five (83.3%) patients had itching, 5 (83.3%) pain, and 4 dyspareunia (66.7%); the other 2 patients did not have sexual intercourse, so the presence or absence of dyspareunia could not be assessed. In no case was a skin biopsy taken, and microbiological samples were obtained in only one case. Among the treatments, pelvic floor physiotherapy was indicated in 5 cases, topical gabapentin in one case, and surgery in one case.

#### Vulvodynia

Two patients were consulted for vulvodynia with a mean ± SD age of 30.66 ± 6.5 years. Both patients had a disease evolution time of >5 years. In no cases were skin biopsies or microbiological samples taken. In terms of treatment, pelvic floor physiotherapy was indicated in one patient, and psychotherapy in another patient.

## Discussion

This study shows the authors’ 5-year experiences as dermatologists managing specific consultations related to diseases of the vulva. At all times, these consultations were organized in close collaboration and with constant communication between the various team members at the gynecology service of our hospital. In all, 148 women were evaluated; those who attended the consultation were most likely to be between 40 and 60 years, and the most frequent diagnosis was lichen sclerosus. This was followed by irritative eczema and lichen simplex chronicus, like other series.^8^, ^9^, ^10^ This type of practice has experienced an increase in the number of patients seen each year, especially in the last 2 years. In fact, in the 2018–2020 period, 89.2% of the women in our series attended a consultation for the first time. On the one hand, these data reflect patients’ increased knowledge and concerns related to the vulva and its different alterations, while on the other hand, the data also represents an increase in the demand for a specialist in dermatology who can act as a reference doctor in the diagnosis and treatment of vulvar diseases. This need also likely emerged as a result of direct and close contact with other specialists such as gynecologists, urologists, and general doctors at our hospital, which is achieved through meetings and bimonthly clinical sessions.

Patients with vulvar diseases often take a long time to seek medical attention, especially because of anxiety and embarrassment. In addition, this delay in going to the doctor causes patients to frequently use home remedies as a treatment, which is frequently associated with the worsening of symptoms.^4^ More than half of the patients in our study, 54.8%, took between 2 and 5 years to consult with us. Given that many of the diseases have a greatly negative impact on patient’s quality of life, and that some of these diseases are pre-neoplastic entities, it is essential to avoid this diagnostic delay by promoting early medical care for patients with vulvar symptoms, thus facilitating an early diagnosis. It is estimated that approximately 1 in 6 women will experience undiagnosed or untreated vulvovaginal discomfort in their lifetime.^11^

The most frequently identified disease process in our series was lichen sclerosus, a chronic inflammatory disease that mainly affects the anogenital area. It most frequently affects prepubertal and postmenopausal women, and its etiology is influenced by genetic and autoimmune factors.^11^ Its true prevalence is unknown and varies depending on whether it is assessed by specialists in gynecology or dermatology.^8^ However, its incidence seems to be increasing and it is one of the most frequent diagnoses in vulvar consultations,^12^ although this could also be the result of an increase in consultations and in the number of diagnoses. In our series, lichen sclerosus represented 41.9% of the total diseases identified, making it the most frequently identified disease, similar to what has been reported in other studies.^8^, ^9^, ^10^ Irritant eczema and lichen simplex chronicus are common dermatoses in the general population that frequently involve the vulva. Among the causes of both dermatoses, it is important to inquire into all products that have been applied topically to the vulva; clinicians should also inquire about intimate hygiene habits, and ask if the patient has been experiencing any type of incontinence.^11^ In cases where there is no improvement, it would be convenient to perform epicutaneous tests to rule out an allergic etiology.^11^ Like lichen sclerosus, these dermatoses represent an important cause of consultation.^9^ The authors detected a single case of allergic contact dermatitis in a patient already diagnosed with a contact allergy to p-phenylenediamine. The delay in the diagnosis of this patient was almost 6 months, as the patient did not associate the use of dye in the hair of the genital area with the discomfort she presented and did not comment on it during consultation. With this specific case, the authors want to highlight the importance of obtaining an exhaustive medical history, which is essential for the diagnosis of cutaneous diseases of the vulva.

Taken together, inflammatory diseases, including lichen sclerosus, vulvar eczema, lichen simplex chronicus, and lichen planus, accounted for 71% of the total series. On the other hand, 8 patients in the series (5.4%) sought consultation for vulvar pain syndromes, including vulvodynia and vestibulodynia. Currently, the prevalence of this condition is unknown in our environment, both due to the lack of patient consultations and the lack of training for those specialists who care for them. Vulvodynia is a frequently occurring, but seldom recognized, the disorder of vulvar pain and is defined as spontaneous or provoked pain in the vulvar area with a duration of >3 months of evolution without an established cause.^11^, ^13^ Although its etiology is multifactorial, it seems that muscle dysfunction of the pelvic floor is a key factor.^14^ When vulvar pain is limited to the vestibule and is generally triggered by friction, it is called secondary provoked vestibulodynia.^15^ In our series, 6 of the 8 patients with vulvar pain were diagnosed with vestibulodynia.

Eleven patients (7.4%) sought consultation for an infectious cause. Contrary to the present study, other authors have stated that the most frequently occurring vulvar diseases in medical consultations are fungal and bacterial infections.^16^ Although it is possible that the most frequent causes of vulvar diseases are infections, most are treated by primary care doctors or referring gynecologists, and these usually do not lead to specific vulva consultations. In fact, other studies similar to ours, and which were carried out by dermatologists, have reported a similar percentage of infections.^8^, ^9^ In the same way, the various inflammatory diseases that involved the vulva – and which were so prevalent in our consultations – included atopic dermatitis, psoriasis, or hidradenitis suppurativa (Fig. 8). These diseases are not typically seen during monographic consultations because dermatologists or other specialists are familiar with their treatment and do not usually refer patients to other specialists. This would explain why lichen sclerosus, a specific pathology of the vulva, is the entity that is most frequently diagnosed.

Due to their particular anatomical characteristics, vulvar diseases are treated differently from other areas of the skin. In inflammatory diseases, the use of ultra-potent topical corticosteroids is essential.^7^ These agents are known to greatly improve symptoms and, in addition, in some diseases such as lichen sclerosus, it seems to reduce the risk of progression to squamous cell carcinoma of the vulva.^11^, ^17^ In fact, it was the most frequently prescribed treatment in our series. A second common option to treat inflammatory diseases of the vulva, and as aligned with current guidelines, includes calcineurin inhibitors.^18^, ^19^ In our opinion, their efficacy is somewhat limited by their frequent side effects of pain and stinging following application. For this reason, in the present study, these agents were only prescribed to 5.4% of patients. On many occasions, patients did not tolerate the creams or commercial preparations that are dispensed in the pharmacy due to patients’ intolerance to the formula or its excipients. Therefore, the formulation of ultra-potent and potent corticosteroids, such as triamcinolone, is essential given their tolerance and application in the genital area.

The authors must highlight the multidisciplinary work in our practice, as we not only work with gynecology, but we integrate a specific pelvic floor unit, as well as specialists in psychology and sexology into our team. Almost 100% of the patients seen reported vulvar pain in relation to the outbreaks of their specific disease, or even in the periods between outbreaks, representing part of the secondary vulvar pain syndromes. In general, vulvar pain can affect up to 20% of women at some point in their lives, and it is known that most cases are associated with pelvic floor disorders.^14^

Pelvic floor physiotherapy is a relatively new discipline that has been gaining relevance in the last decade, not only due to its effectiveness as a treatment for women with primary or secondary vulvar pain, but also given that it aids in the prevention and maintenance of pelvic floor health during pregnancy, postpartum, and climacteric.^14^, ^20^

The great majority of patients attended in our department referred to vulvar pain or discomfort. Pelvic floor physiotherapy allows an assessment of these processes and early diagnosis and treatment of possible secondary vulvodynia and muscle pelvic dysfunctions.

Unfortunately, in our environment, neither the public health system nor insurance companies typically cover this type of assistance; so, although the authors recommend a large percentage of patients come in for an assessment, the economic limitations and lack of information on this specialty mean that only a small percentage attend (23% of the total), with clear improvements in all of them. Something similar has occurred with psychotherapy and sexology. A large percentage of women (45.9%) reported pain or discomfort with sexual intercourse, but only two patients consulted with a specialist in psychology and sexology. Financial reasons, shame, and the fact that consultation with a psychologist/sexologist is considered socially taboo were the main cited reasons why patients abstained from consulting. In future studies, the authors consider starting from baseline studies like this one to assess how pelvic floor physiotherapy and psychotherapy influence the quality of life of patients with vulvar diseases.

The present study has several limitations. On the one hand, this study featured the various limitations associated with retrospective studies. On the other, the limited number of patients, as well as the presence of certain entities, such as vulvar diseases with an infectious cause, are usually managed in primary care without monographic consultations; therefore, these cases were not included in this study. Furthermore, no data were collected on the response and/or satisfaction of patients with the different treatments. Finally, the fact that all patients came from a single private hospital limited the possibility with which relevant conclusions could be drawn as, when compared with the public health system, is remarkably easier to get a consultation with a Dermatologist and other medical specialties and health-related services. On the other hand, there are different socioeconomic limitations associated with access to private health care and the different insurance coverages.

## Conclusion

In conclusion, the authors present their experience after 5 years of carrying out specific vulva consultations. Vulvar diseases are frequently occurring and often associated with high morbidity. It is thus essential that these patients are evaluated in specific units that guarantee comprehensive care. It is also necessary for these units to include other specialists with experience in the field, such as gynecologists, urologists, physiotherapists, or psychologists, who can ensure a multidisciplinary approach to providing care to patients with vulvovaginal symptoms.

## Financial support

None declared.

## Authors’ contributions

Fernando García-Souto: Preparation and writing of the manuscript; data collection, analysis and interpretation; statistical analysis; critical literature review.

Ana Isabel Lorente-Lavirgen: Study conception and planning; preparation and writing of the manuscript; intellectual participation in propaedeutic and/or therapeutic management of studied cases; approval of the final version of the manuscript.

Francisco Manuel Ildefonso Mendonça: Intellectual participation in propaedeutic and/or therapeutic management of studied cases; critical literature review; approval of the final version of the manuscript.

Manuel García-de-Lomas: Intellectual participation in propaedeutic and/or therapeutic management of studied cases; critical literature review.

Mariana Viktoria Hoffner-Zuchelli: Intellectual participation in propaedeutic and/or therapeutic management of studied cases; critical literature review.

Desiree Rodriguez-Ojeda: Intellectual participation in propaedeutic and/or therapeutic management of studied cases.

Elena Pozo: Intellectual participation in propaedeutic and/or therapeutic management of studied cases; Critical literature review.

José Bernabéu-Wittel: Intellectual participation in propaedeutic and/or therapeutic management of studied cases; Approval of the final version of the manuscript.

## Conflict of interest

None declared.

## References

[1] Pathak D., Agrawal S., Dhali T.K. (2011). Prevalences of and risk factors for vulvar diseases in Nepal: a hospital-based study. Int J Dermatol.

[2] Hickey S., Bell H. (2010). Quality of life in the vulvar clinic: a pilot study. J Low Genit Tract Dis.

[3] Lawton S., Littlewood S. (2013). Vulval skin conditions: disease activity and quality of life. J Low Genit Tract Dis.

[4] Mauskar M.M., Marathe K., Venkatesan A., Schlosser B.J., Edwards L. (2020). Vulvar diseases: approach to the patient. J Am Acad Dermatol.

[5] Venkatesan A., Farsani T., O’Sullivan P., Berger T. (2012). Identifying competencies in vulvar disorder management for medical students and residents: a survey of US vulvar disorder experts. J Low Genit Tract Dis.

[6] Edwards L. (2010). Preface: vulvovaginal dermatology. Dermatol Clin.

[7] Barchino-Ortiz L., Suárez-Fernández R., Lázaro-Ochaita P. (2012). Dermatosis inflamatorias vulvares. Actas Dermosifiliogr.

[8] Goncalves D.L.M., Romero R.L., Ferreira P.L., Santi C.G. (2019). Clinical and epidemiological profile of patients attended in a vulvar clinic of the dermatology outpatient unit of a tertiary hospital during a 4-year period. Int J Dermatol.

[9] Anemüller W., Recke A., Altgassen C., Kelling K. (2012). Aufbau einer interdisziplinären Vulvasprechstunde. JDDG - J Ger Soc Dermatol.

[10] Sullivan A.K., Straughair G.J., Marwood R.P., Staughton R.C., Barton S.E. (1999). A multidisciplinary vulva clinic: the role of genito-urinary medicine. J Eur Acad Dermatol Venereol.

[11] Mauskar M.M., Marathe K., Venkatesan A., Schlosser B.J., Edwards L. (2020). Vulvar diseases: conditions in adults and children. J Am Acad Dermatol.

[12] Bleeker M.C.G., Visser P.J., Overbeek L.I.H., Beurden M., Berkhof J. (2016). Lichen sclerosus: incidence and risk of vulvar squamous cell carcinoma. Cancer Epidemiol Biomarkers Prev.

[13] Pukall C.F., Goldstein A.T., Bergeron S., Foster D., Stein A., Kellogg-Spadt S. (2016). Vulvodynia: definition, prevalence, impact, and pathophysiological factors. J Sex Med.

[14] Prendergast SA (2017). Pelvic floor physical therapy for vulvodynia: a clinician’s guide. Obstet Gynecol Clin North Am.

[15] Bautrant E., Porta O., Murina F., Mühlrad H., Levêque C., Riant T. (2019). Provoked vulvar vestibulodynia: Epidemiology in Europe, physio-pathology, consensus for first-line treatment and evaluation of second-line treatments. J Gynecol Obstet Hum Reprod.

[16] Bauer A., Greif C., Vollandt R., Merker A., Elsner P. (1999). Vulval diseases need an interdisciplinary approach. Dermatology.

[17] Lee A., Fischer G. (2018). Diagnosis and treatment of vulvar lichen sclerosus: an update for dermatologists. Am J Clin Dermatol.

[18] Lewis F.M., Tatnall F.M., Velangi S.S., Bunker C.B., Kumar A., Brackenbury F. (2018). British Association of Dermatologists guidelines for the management of lichen sclerosus, 2018. Br J Dermatol.

[19] van der Meijden W.I., Boffa M.J., Harmsel W.A.T., Kirtschig G., Lewis F.M., Moyal-Barracco M. (2017). 2016 European guideline for the management of vulval conditions. J Eur Acad Dermatol Venereol.

[20] Schreiner L., Crivelatti I., Oliveira J.M., Nygaard C.C., Santos T.G. (2018). Systematic review of pelvic floor interventions during pregnancy. Int J Gynecol Obstet.

